# Histologic chorioamnionitis and fat mass accretion in infants born preterm

**DOI:** 10.1038/s41390-025-04413-2

**Published:** 2025-09-19

**Authors:** Emily Gunawan, Viral G. Jain, Shakia Hardy, M. Ryan Irvin, Ariel A. Salas

**Affiliations:** 1 Department of Pediatrics, University of Alabama at Birmingham, Birmingham, AL USA; 2 School of Public Health, University of Alabama at Birmingham, Birmingham, AL USA

## Abstract

**Background:**

In utero insults such as chorioamnionitis are associated with adverse outcomes. This study aims to examine the association between chorioamnionitis and fat mass (FM) in very preterm infants.

**Methods:**

We conducted a retrospective cohort study of mother-infant dyads born <32^6/7^ weeks of gestation. Infant FM accretion was measured using air displacement plethysmography at term-equivalent age. Histological chorioamnionitis severity was staged based on placental pathology and included maternal/chorion-amnion inflammatory response (MIR) and fetal/umbilical cord inflammatory response (FIR). The association between chorioamnionitis severity and FM accretion was analyzed using linear regression models and mediation analyses.

**Results:**

Among 375 mother-infant dyads analyzed, 104 (28%) dyads had MIR. FIR was found in 85 dyads with MIR (82%). Infants without MIR had lower FM in Kg and lower FM z scores than those with MIR (*p* = 0.0001). Infants with severe MIR had higher body fat percentages (Stage 3: 18% vs Stage 1: 14%, *p* < 0.0001). There were no significant differences in other anthropometric growth rates. Gestational age partially mediated this association (49%).

**Conclusion:**

Severe histological chorioamnionitis is associated with greater FM accretion at term-equivalent age, independent of gestational age. Without long-term data, it remains unclear whether this early-onset effect is transient or persists into later childhood.

**Impact statement:**

- Chorioamnionitis is common in infants born preterm and is strongly associated with preterm birth.
- Infants born preterm exposed to chorioamnionitis have an increased risk of abnormal fat mass accretion at term equivalent age.
- Accounting for the severity of chorioamnionitis could improve the interpretation of body fat accretion in infants born preterm.

## Introduction

The placenta is a vital organ that mediates the complex interactions between maternal and fetal physiology to facilitate optimum nutrient exchange for the growing fetus.^1^ The placental morphology and function continuously evolve and adapt throughout its relatively short lifespan. Pathologies found in the placenta may help reveal in-utero insults and inform postnatal care.^2–5^ One such pathology is histologic chorioamnionitis (HCA), an inflammatory condition that can occur without any clinical signs and symptoms.^6^ HCA is a common cause of preterm birth and accounts for up to 40–80% of premature deliveries. Exposure to HCA may injure the developing organs of infants born preterm. In preterm infants, HCA is associated with a higher risk of short-term adverse outcomes,^7,8^ including sepsis,^9^ respiratory disorders,^10–12^ neurodevelopment impairment,^8,13–16^ necrotizing enterocolitis,^17^ and death.^6,18,19^

In utero insults such as HCA have also been associated with long-term metabolic disorders.^18^ However, the underlying mechanisms remain unclear. One possible pathway by which HCA might exert lasting effects involves early alterations in body composition. Greater fat mass (FM) accretion from birth to early childhood has been associated with higher fasting glucose and insulin levels in children at 5 years of age.^20^ Infants born preterm have significantly greater FM at term-equivalent age than their term-born counterparts, and unlike fat-free mass accretion, FM accretion in preterm infants appears to be independent of nutritional intake.^21,22^ Since FM accretion appears essential for fluid balance and thermoregulation in preterm infants, its accumulation may represent an adaptive response to the abrupt transition from intrauterine to extrauterine life. In this context, it is possible that HCA could influence FM accretion by modulating this adaptive response. We hypothesized that the association between HCA and FM accretion at term-equivalent age is dose-dependent and independent of gestational age.

## Methods

### Study design, setting, and participants

We conducted a retrospective cohort study to determine the association between HCA and FM accretion in infants born preterm and delineate the role of early preterm delivery on FM accretion using prospectively collected data of mother-infant dyads born <32^6/7^ weeks of gestation admitted to the tertiary neonatal intensive care unit (NICU) at the University of Alabama at Birmingham (UAB) between 2016 and 2022. Mother-infant dyads with placental pathology reports and body composition outcomes at term equivalent age were included. Infants with genetic abnormalities were excluded from the analysis. Waiver of authorization and informed consent were obtained from the UAB Institutional Review Board (IRB-300009890).

### Growth outcome measures

The primary outcome was FM accretion, defined by FM in kilograms and body fat percentage (%BF) measured using air displacement plethysmography (ADP) at 36 weeks postmenstrual age (PMA) or discharge, whichever occurred first. To compare FM and %BF between groups, we calculated FM-for-age and %BF-for-age *z* scores using the LMS method.^23^

Secondary outcomes were mid-upper arm circumference (MUAC) at 36 weeks PMA or discharge, which corresponds to term-equivalent age, and anthropometric growth rates spanning from birth to 36 weeks PMA or discharge. These growth rates were reported as changes in weight-for-age, length-for-age, and head circumference-for-age *z* scores from birth to 36 weeks PMA or discharge. Weight gain in g/kg/day from birth to 36 weeks PMA or discharge was calculated using the 2-point average and exponential methods.^24^

### HCA severity

Our primary exposure was the severity of HCA based on placental pathology reports. Pathologic examination of the placenta is routinely performed in cases of preterm delivery at our institution. Depending on the location of inflammation, HCA is separated into maternal inflammatory response (MIR) and fetal inflammatory response (FIR), which corresponds with funisitis. Specifically, MIR occurs in the chorion and amnion while FIR occurs in the umbilical cord.^25^ The severity of each was categorized according to the staging criteria proposed by the Society for Pediatric Pathology.^26^ Clinical chorioamnionitis cases were included and staged accordingly based on histologic findings of placental inflammation.

### Data collection

All infant and maternal variables were derived from the respective medical records, while placental variables were obtained from placental pathology reports. The following neonatal variables were examined: birth weight, sex, small for gestational age, birth weight *z* score, race, delivery mode, any antibiotics administered at ≤72 h prior to delivery regardless of the indication, and antenatal steroid treatment doses. Race was classified as “Black” and “Non-Black”. We categorized antenatal steroid treatment doses as “None”, “1 dose”, and “≥2 doses”. Maternal variables assessed were age, body mass index (BMI), smoking status, substance use, as well as hypertension, and diabetes mellitus (DM) based on diagnosis codes. Due to insufficient data on pre-pregnancy BMI or BMI at first obstetric visit, maternal BMI at hospital admission was used and categorized with a threshold of 30.0 kg/m^2^. We included all types of DM, namely gestational diabetes, Type 1 DM, and Type 2 DM. Placental variables evaluated included placental weight and disk dimensions (length and width).

### Statistical analysis

Descriptive statistics were presented using frequencies and percentages for categorical data. Continuous data were summarized with means and standard deviations or medians with interquartile ranges. We summarized the demographic characteristics and growth outcomes by HCA severity. We evaluated differences in demographic characteristics and growth outcomes by HCA severity using one-way ANOVA with Tukey–Kramer’s HSD for the post-hoc t-test or Kruskal–Wallis and Chi-square tests. The association between HCA and growth outcomes was analyzed using linear regression models. We adjusted models for significant confounders (i.e., variables resulting in a 10% change in the relationship between HCA and growth outcomes). While both birth weight and birth weight *z* score were significant confounders, birth weight *z* score was chosen to account for fetal growth restriction. Our final model included significant confounders birth weight *z* score, delivery mode, maternal BMI, and hypertension. Due to the possibility of unequal placental sharing among dyads with multiple gestations, we conducted a sensitivity analysis that only included mother-infant dyads with a singleton birth. All statistical analyses were performed using SAS 9.4 (SAS Institute Inc., Cary, NC) and were performed at *α* = 0.05 significance level.

As preterm birth lies on the causal pathway, we investigated whether gestational age mediated the relationship between HCA and growth. Mediation analysis was conducted using the Valeri and VanderWeele SAS macro.^27^

## Results

There were 1180 mother-infant dyads born <32^6/7^ weeks of gestation admitted to UAB between 2016 and 2022. Of these, we excluded 804 dyads that did not have body composition measurements at approximately term-equivalent age and 1 dyad with no placental pathology report. Our final analytic sample consisted of 375 mother-infant dyads with slightly more Black infants (53%) and more female infants (55%). The mean birth weight was 1239 ± 405 g, and the median gestational age was 29 weeks (Table 1). Basic nutritional data were available for 357 infants. The median time to initiation of enteral feeds was 2 days (IQR: 2–3), time to reach full feeds was 8 days (IQR: 6–9), and fortification was initiated at a median of 15 days (IQR: 12–19). Approximately 70% of infants received predominantly human milk diets during the first 28 days. None of these feeding characteristics differed significantly across inflammatory response groups.

**Table 1:** Demographic characteristics of mother-infant dyads by acute placental inflammation (API) severity.

	Total (*n* = 376)	MIR none (*n* = 271)	MIR stage 1 (*n* = 19)	MIR stage 2 (*n* = 58)	MIR stage 3 (*n* = 28)	*p* value
Neonatal variables						
Birth Weight, g	1239 ± 405^a^	1268 ± 405^a^	1432 ± 375^a^	1192 ± 369^a^	931 ± 334^a^	**<0.0001**
Gestational age, weeks	29 (27–31)^b^	30 (28–31)^b^	30 (28–32)^b^	28 (26–30)^b^	26 (25–27)^b^	**<0.0001**
Small for gestational age, *n*(%)	35 (9)	33 (12)	0 (0)	1 (2)	1 (4)	**0.0229**
Birth weight *z* score	–0.09 ± 0.86^a^	−0.25 ± 0.86^a^	0.46 ± 0.62^a^	0.28 ± 0.69^a^	0.30 ± 0.92^a^	**<0.0001**
Sex, *n*(%)						0.2851
Female	207 (55)	151 (56)	8 (42)	29 (50)	19 (68)	
Male	169 (45)	120 (44)	11 (58)	29 (50)	9 (32)	
Race, *n*(%)						0.0688
Black	198 (53)	134 (49)	14 (74)	31 (53)	19 (68)	
Non-black^c^	178 (47)	137 (51)	5 (26)	27 (47)	9 (32)	
Multiple gestation, *n*(%)						**0.0106**
Yes	100 (27)	84 (31)	4 (21)	6 (10)	6 (21)	
No	276 (73)	187 (69)	15 (79)	52 (90)	22 (79)	
Delivery mode, *n*(%)						**<0.0001**
Vaginal	130 (35)	64 (24)	13 (68)	38 (66)	15 (54)	
C-section	246 (65)	207 (76)	6 (32)	20 (34)	13 (46)	
Antibiotics prior to delivery, *n*(%)						**<0.0001**
Yes	270 (72)	178 (66)	13 (68)	53 (91)	26 (93)	
No	106 (28)	93 (34)	6 (32)	5 (9)	2 (7)	
Antenatal steroids, *n*(%)						**0.0316**
None	25 (7)	18 (7)	4 (21)	2 (3)	1 (4)	
1 dose	57 (15)	39 (14)	6 (32)	7 (12)	5 (18)	
≥2 doses	294 (78)	214 (79)	9 (47)	49 (85)	22 (78)	
Maternal variables						
Age, years	28 ± 6^a^	28 ± 6^a^	27 ± 9^a^	28 ± 6^a^	29 ± 6^a^	0.7690
BMI at hospital admission ≥ 30.0 kg/m^2^, *n*(%)						**0.0153**
Yes	244 (66)	190 (71)	9 (50)	28 (52)	17 (61)	
No	123 (34)	44 (29)	9 (50)	26 (48)	11 (39)	
Smoking status, *n*(%)						0.2521
Yes	53 (14)	40 (15)	3 (16)	4 (7)	6 (23)	
No	314 (86)	226 (85)	16 (84)	52 (93)	20 (77)	
Substance use, *n*(%)						0.3373
Yes	40 (11)	28 (11)	4 (21)	4 (7)	4 (15)	
No	325 (89)	236 (89)	15 (79)	53 (93)	22 (85)	
Hypertension, *n*(%)						**<0.0001**
Yes	230 (61)	202 (75)	3 (16)	13 (22)	12 (43)	
No	146 (39)	69 (25)	16 (84)	45 (78)	16 (57)	
Diabetes, *n*(%)						0.4753
Yes	71 (19)	53 (20)	1 (5)	11 (19)	6 (21)	
No	305 (81)	218 (80)	18 (95)	47 (81)	22 (79)	
Placental variables						
Weight, g	243 ± 84^a^	238 ± 84^a^	284 ± 64^a^	263 ± 90^a^	225 ± 68^a^	**0.0158**
Disk dimensions, cm						
Length	15.6 ± 2.3^a^	15.6 ± 2.4^a^	16.2 ± 1.3^a^	15.5 ± 2.3^a^	15.5 ± 2.3^a^	0.7383
Width	12.6 ± 2.5^a^	12.5 ± 2.5^a^	13.6 ± 2.4^a^	12.9 ± 2.3^a^	11.8 ± 2.6^a^	0.0684

*MIR* maternal inflammatory response.

^a^Mean ± SD.

^b^Median (IQR).

^c^“Non-Black” races include White, Asian, and Hispanic ethnicity.

Bold text denotes statistical significance *p* < 0.05.

We identified 104 (28%) dyads with MIR, indicating HCA. Funisitis was present in 82% of MIR cases. Birth weight, gestational age, multiple gestation, delivery mode, antibiotics administered prior to delivery, antenatal steroid treatment doses, maternal obesity, hypertension, and placental weight differed significantly by HCA severity (Table 1). Infants with stage 3 MIR had lower birth weight, had smaller gestational age, received antibiotics treatment prior to delivery, and had lower placental weight compared to those without MIR and those with lower MIR stages (Table 1). Conversely, compared to those with MIR, mothers without MIR were more likely to have obesity, hypertension, and Cesarean section delivery (Table 1). There were no significant differences in sex, race, maternal age, maternal smoking status, maternal substance use, maternal diabetes, and placental length and width by HCA severity.

We observed statistically significant differences in FM accretion, MUAC, and change in weight *z* score by HCA severity (Table 2). At term-equivalent age, infants without MIR had lower FM (0.32 kg vs Stage 3: 0.45 kg, *p* < 0.0001) and lower FM *z* score (0.63 ± 1.10 vs Stage 3: 1.34 ± 1.05, *p* = 0.0001) than those with MIR (Table 2). Infants with severe MIR had higher %BF (Stage 3: 18.3% vs Stage 1: 14.0%, *p* < 0.0001) and % BF *z* score (Stage 3: 2.08 ± 1.00 vs Stage 1: 1.17 ± 1.27, *p* = 0.0001) than those with Stage 1 MIR and those without MIR (Table 2). There were no significant differences in other anthropometric growth rates by HCA severity. Significant differences in FM accretion persisted after excluding growth-restricted infants from the analyses (Supplemental Tables S1 and S2).

**Table 2:** Growth outcomes by maternal inflammatory response (MIR).

	Total (*n* = 376)	MIR none (*n* = 271)	MIR stage 1 (*n* = 19)	MIR stage 2 (*n* = 58)	MIR stage 3 (*n* = 28)	*p* value
Body composition at term-equivalent age^a^						
Fat mass, kg	0.33 (0.24–0.44)^b^	0.32 (0.23–0.42)^b^	0.34 (0.23–0.46)^b^	0.37 (0.29–0.45)^b^	0.45 (0.34–0.66)^b^	**<0.0001**
Body fat percentage, %	14.9 ± 4.8^c^	14.4 ± 4.8^c^	14.0 ± 5.5^c^	16.2 ± 4.1^c^	18.3 ± 4.4^c^	**<0.0001**
Fat mass *z* score	0.78 ± 1.10^c^	0.63 ± 1.10^c^	0.84 ± 1.11^c^	1.18 ± 0.93^c^	1.34 ± 1.05^c^	**0.0001**
Body fat percentage *z* score	1.39 ± 1.17^c^	1.25 ± 1.17^c^	1.17 ± 1.27^c^	1.77 ± 1.01^c^	2.08 ± 1.00^c^	**0.0001**
Mid-upper arm circumference, cm	8.8 (8.0–9.3)^b^	8.6 (8.0–9.1)^b^	8.8 (8.3–9.0)^b^	8.8 (8.0–9.3)^b^	10.0 (9.0–10.3)^b^	**0.0353**
Anthropometric growth rates^d^						
Change in weight *z* score	−0.95 ± 0.59^c^	−0.90 ± 0.55^c^	−0.91 ± 0.59^c^	−1.02 ± 0.58^c^	−1.31 ± 0.87^c^	**0.0030**
Change in length *z* score	−1.29 ± 0.93^c^	−1.23 ± 0.84^c^	−1.36 ± 1.10^c^	−1.44 ± 0.96^c^	−1.59 ± 1.38^c^	0.1189
Change in head circumference *z* score	−0.85 (−1.39 to −0.24)^b^	−0.84 (−1.41 to −0.22)^b^	−0.65 (−1.01 to 0.17)^b^	−0.82 (−1.30 to −0.19)^b^	−1.14 (−1.80 to −0.73)	0.0618
Weight gain, g/day	20 (16–24)^b^	20 (16–24)^b^	23 (14–26)^b^	21 (17–25)^b^	19 (14–23)^b^	0.4883
Weight gain (2-point), g/kg/day	13 (10–14)^b^	13 (10–14)^b^	12 (10–14)^b^	12 (11–14)^b^	13 (12–14)^b^	0.6988
Weight gain (Exponential), g/kg/day	13 (11–15)^b^	13 (10–15)^b^	12 (10–14)^b^	13 (12–15)^b^	14 (12–15)^b^	0.3648
Length gain, cm/week	0.8 (0.6–1.0)^b^	0.8 (0.6–1.0)^b^	0.8 (0.3–1.0)^b^	0.8 (0.6–1.1)^b^	0.8 (0.6–1.0)^b^	0.8672
Head Circumference gain, cm/week	0.7 (0.5–0.8)^b^	0.7 (0.5–0.8)^b^	0.6 (0.6–0.9)^b^	0.7 (0.6–0.8)^b^	0.7 (0.7–0.8)^b^	0.1434

^a^Measured at 36 weeks post-menstrual age (PMA) or discharge.

^b^Median (IQR).

^c^Mean ± SD.

^d^Calculated between birth and 36 weeks PMA or discharge.

Bold text denotes statistical significance *p* < 0.05.

### The association between MIR and growth outcomes

Our unadjusted model revealed that with each stage of MIR progression, FM at term-equivalent age increased by 0.05 kg, %BF increased by 1.1%, and MUAC increased by 0.2 cm (Table 3). MIR progression was also positively associated with FM and %BF *z* scores at term-equivalent age but negatively associated with a change in weight *z* score from birth to 36-week PMA (Table 3).

**Table 3:** Association between maternal inflammatory response (MIR) and growth outcomes.

	Model 1			Model 2		
	*β* (95% CI)	*β*^a^	*p* value	*β* (95% CI)	*β*^a^	*p* value
Body composition at term-equivalent age^a^						
Fat mass, kg	0.05 (0.03–0.07)	0.22	**<0.0001**	0.03 (0.01–0.05)	0.14	**0.0104**
Body fat percentage, %	1.1 (0.6–1.6)	0.2	**<0.0001**	1.1 (0.5–1.6)	0.2	**<0.0001**
Fat mass *z* score	0.25 (0.15–0.36)	0.23	**<0.0001**	0.17 (0.05–0.28)	0.15	**0.0043**
Body fat percentage *z* score	0.26 (0.14–0.38)	0.22	**<0.0001**	0.26 (0.13–0.39)	0.22	**<0.0001**
Mid-upper arm circumference, cm	0.2 (0.1–0.4)	0.2	**0.0168**	0.1 (−0.1 to 0.3)	0.1	0.4491
Anthropometric growth rate^b^						
Change in Weight z score	−0.10 (−0.16 to −0.04)	−0.17	**0.0007**	−0.07 (−0.13 to −0.01)	−0.11	**0.0483**

*β* = Unstandardized coefficient; β* = Standardized coefficient.

Model 1 = Crude.

Model 2 = Adjusted for birth weight *z* score, delivery mode, maternal BMI at hospital admission ≥30.0 kg/m,^2^ and hypertension.

^a^Measured at 36 weeks post-menstrual age (PMA) or discharge.

^b^Calculated between birth and 36 weeks PMA or discharge.

Bold text denotes statistical significance *p* < 0.05.

After adjustment for birth weight *z* score, delivery mode, maternal BMI, and hypertension, the association between MIR and FM as well as %BF persisted. (Table 3). With each stage of MIR progression, FM at term-equivalent age increased by 0.03 kg and %BF increased by 1.1% (Table 3). MIR progression remained positively associated with FM and %BF *z* scores at term-equivalent age but negatively associated with change in weight *z* score from birth to 36-week PMA (Table 3). Sensitivity analyses restricted to mother-infant dyads with singleton birth showed a similar magnitude of association between MIR and various growth outcomes (Table 4).

**Table 4:** Association between maternal inflammatory response (MIR) and growth outcomes restricted to mother-infant dyads with singleton birth (*n* = 275).

	Model 1			Model 2		
	*β* (95% CI)	*β*^a^	*p* value	*β* (95% CI)	*β*^a^	*p* value
Body composition at term-equivalent age^a^						
Fat mass, kg	0.05 (0.03–0.07)	0.25	**<0.0001**	0.03 (0.01–0.06)	0.17	**0.0167**
Body fat percentage, %	1.1 (0.5–1.6)	0.2	**<0.0001**	1.0 (0.4–1.6)	0.2	**0.0022**
Fat mass *z* score	0.25 (0.12–0.37)	0.23	**<0.0001**	0.09 (−0.04 to 0.22)	0.09	0.1745
Body fat percentage *z* score	0.25 (0.11–0.38)	0.22	**0.0003**	0.19 (0.04–0.35)	0.17	**0.0141**
Mid-upper arm circumference, cm	0.23 (0.04–0.42)	0.18	**0.0201**	0.06 (−0.16 to 0.28)	0.05	0.5678
Anthropometric growth rate^b^						
Change in Weight *z* score	−0.13 (−0.20 to −0.06)	−0.22	**0.0002**	−0.09 (−0.17 to −0.01)	−0.16	**0.0209**

*β* = Unstandardized coefficient; β* = Standardized coefficient.

Model 1 = Crude/Unadjusted.

Model 2 = Adjusted for birth weight *z* score, delivery mode, maternal BMI at hospital admission ≥30.0 kg/m,^2^ and hypertension.

^a^Measured at 36 weeks post-menstrual age (PMA) or discharge.

^b^Calculated between birth and 36 weeks PMA or discharge.

Bold text denotes statistical significance *p* < 0.05.

As birth weight *z* score is a function of gestational age, our mediation models were adjusted for significant baseline confounders, excluding birth weight *z* score. We observed that gestational age significantly but only partially (49%) mediated the association between MIR and FM *z* score at term-equivalent age (indirect effect of gestational age on FM z score: *β* = 0.12, 95% CI: 0.05–0.19; direct effect of MIR on FM *z* score: *β* = 0.12, 95% CI: 0.01–0.25) adjusted for significant confounders (Supplemental Table S3). Therefore, almost half of the total relationship between MIR and FM *z* score was indirectly mediated by gestational age, which includes the downstream effects of fetal growth restriction as a function of gestational age, whereas MIR directly contributed to the other half. Gestational age partially mediated the association between MIR and %BF (88%), %BF *z* score (58%), and change in weight *z* score (59%), although their respective direct coefficients were not significant.

### Funisitis

Infants without FIR had significantly lower FM (0.33 kg vs Stage 2: 0.39 kg, *p* = 0.0076), %BF (14.8% vs Stage 1: 16.8%, *p* = 0.0182), FM z score (0.66 ± 1.10 vs Stage 3: 1.23 ± 0.80, *p* = 0.0041) and %BF *z* scores (1.27 ± 1.16 vs Stage 1: 1.77 ± 1.34, *p* = 0.0138) at term-equivalent age compared to those with FIR (Table 5). On multivariate linear regression analyses adjusted for the same confounders, FIR progression remained associated with increasing %BF and positively associated with FM and %BF *z* scores (Table 6). Gestational age partially mediated the association between FIR and FM *z* score (41%) and %BF *z* score (50%), although their direct effects were not significant (Supplemental Table S4).

**Table 5:** Growth outcomes by fetal inflammatory response (FIR).

	Total (*n* = 371)	FIR none (*n* = 286)	FIR stage 1 (*n* = 29)	FIR stage 2 (*n* = 41)	FIR stage 3 (*n* = 15)	*p* value
Body composition at term-equivalent age^a^						
Fat mass, kg	0.33 (0.24–0.44)^b^	0.33 (0.23–0.42)^b^	0.39 (0.29–0.55)^b^	0.39 (0.32–0.49)^b^	0.33 (0.29–0.46)^b^	**0.0076**
Body fat percentage, %	15.2 (12.2–17.5)^b^	14.8 (11.9–17.2)^b^	16.8 (13.4–19.3)^b^	16.3 (14.1–19.1)^b^	15.2 (12.7–18.3)^b^	**0.0182**
Fat mass *z* score	0.77 ± 1.10^c^	0.66 ± 1.10^c^	1.18 ± 1.16^c^	1.10 ± 1.05^c^	1.23 ± 0.80^c^	**0.0041**
Body fat percentage *z* score	1.37 ± 1.17^c^	1.27 ± 1.16^c^	1.77 ± 1.34^c^	1.70 ± 1.04^c^	1.76 ± 0.88^c^	**0.0138**
Mid-upper arm circumference, cm	8.8 ± 1.3^c^	8.7 ± 1.4^c^	9.3 ± 1.3^c^	8.9 ± 1.1^c^	8.8 ± 0.9^c^	0.1988
Anthropometric growth rates^a^						
Change in weight *z* score	−0.95 ± 0.59^c^	−0.91 ± 0.56^c^	−1.10 ± 0.60^c^	−1.00 ± 0.73^c^	−1.19 ± 0.64^c^	0.1081
Change in length *z* score	−1.28 ± 0.92^c^	−1.24 ± 0.88^c^	−1.75 ± 0.84^c^	−1.23 ± 1.10^c^	−1.26 ± 1.04^c^	**0.0382**
Change in head circumference *z* score	−0.82 ± 1.02^c^	−0.78 ± 1.02^c^	−0.90 ± 0.79^c^	−1.02 ± 1.19^c^	−0.87 ± 0.94^c^	0.5211
Weight gain, g/day	20 ± 6^c^	20 ± 6^c^	20 ± 7^c^	21 ± 7^c^	20 ± 5^c^	0.9691
Weight gain (2-point), g/kg/day	12 (10–14)^b^	13 (10–14)^b^	12 (11–13)^b^	12 (12–14)^b^	12 (10–13)^b^	0.5493
Weight gain (Exponential), g/kg/day	13 (11–15)^b^	13 (10–15)^b^	13 (12–14)^b^	13 (12–15)^b^	13 (10–14)^b^	0.4653
Length gain, cm/week	0.8 ± 0.4^c^	0.8 ± 0.4^c^	0.8 ± 0.3^c^	0.8 ± 0.6^c^	0.8 ± 0.4^c^	0.9159
Head circumference gain, cm/week	0.7 (0.5–0.8)^b^	0.7 (0.5–0.8)^b^	0.7 (0.6–0.8)^b^	0.7 (0.6–0.8)^b^	0.7 (0.6–0.9)^b^	0.3757

^a^Measured at 36 weeks post-menstrual age (PMA) or discharge.

^b^ Median (IQR).

^c^Mean ± SD.

^d^Calculated between birth and 36 weeks PMA or discharge.

Bold text denotes statistical significance *p* < 0.05.

**Table 6:** Association between fetal inflammatory response (FIR) and growth outcomes.

	Model 1			Model 2		
	*β* (95% CI)	*β*^a^	*p* value	*β* (95% CI)	*β*^a^	*p* value
Body composition at term-equivalent age^a^						
Fat mass, kg	0.04 (0.01–0.06)	0.15	**0.0045**	0.02 (−0.01 to 0.05)	0.08	0.1363
Body fat percentage, %	0.8 (0.3–1.4)	0.1	**0.0046**	0.8 (0.1–1.4)	0.1	**0.0155**
Fat mass *z* score	0.23 (0.09–0.36)	0.17	**0.0009**	0.14 (0.01–0.27)	0.10	**0.0394**
Body fat percentage *z* score	0.21 (0.07–0.35)	0.15	**0.0032**	0.20 (0.05–0.35)	0.14	**0.0094**
Anthropometric growth rate^b^						
Change in length *z* score	−0.03 (−0.14 to −0.08)	−0.03	0.5652	N/A	N/A	N/A

*β* = Unstandardized coefficient; β* = Standardized coefficient.

Model 1 = Crude/Unadjusted.

Model 2 = Adjusted for birth weight z score, delivery mode, maternal BMI at hospital admission ≥30.0 kg/m^2^, and hypertension.

^a^Measured at 36 weeks post-menstrual age (PMA) or discharge.

^b^Calculated between birth and 36 weeks PMA or discharge.

Bold text denotes statistical significance *p* < 0.05.

## Discussion

In this retrospective cohort study of mother-preterm infant dyads, we found a dose-dependent association between HCA severity and FM accretion. Each stage of MIR progression was directly associated with an increase in FM at term-equivalent age by 30 grams and %BF by 1.1%. Each stage of FIR progression was directly associated with an increase in %BF at term-equivalent age by 0.8%. We also found that gestational age only partially mediated the association between HCA severity and growth outcomes.

While HCA appears to contribute to a small, short-term increase in FM accretion at term-equivalent age, it is important to consider that recent evidence suggests preterm infants tend to exhibit adiposity and metabolic health profiles increasingly similar to those of term-born peers as they age.^28–30^ Without long-term follow-up data, our findings can only suggest a potential link between HCA, alterations in lipid metabolism, and increased risk of metabolic disorders in preterm infants. Determining the specific contributions of preterm birth and HCA to long-term metabolic outcomes is challenging, particularly given the limited number of longitudinal studies capable of disentangling the complex causal pathways involved.^31^

Prior studies have demonstrated that HCA progression is associated with systemic inflammation and higher cortisol levels in the first postnatal week in preterm infants exposed.^18,32,33^ It is plausible that these mechanisms induce fat accumulation in these infants. Cortisol has been shown to promote visceral fat tissue deposition.^34^ Visceral fat has high metabolic activity and contributes to systemic inflammation.^35^ Meanwhile, systemic inflammation potentially alters insulin-signaling pathways and causes insulin resistance, which promotes further fat accumulation in general and perpetuates the cycle as observed in individuals with obesity.^36^

A smaller study of 88 matched very preterm infants by González et al. found that HCA did not affect weight gain, changes in weight *z* scores, or extrauterine growth restriction when adjusted for gestational age, IUGR, and neonatal complications.^37^ Conversely, we found that the severity of HCA was negatively associated with a change in weight *z* score at 36 weeks PMA or discharge. The association was partially (59%) mediated by gestational age, although a direct effect was not significant. Our study also found that HCA did not affect changes in length and head z scores or postnatal weight, length, and head circumference gains, which have not been reported previously. Uthaya et al. found that preterm infants had significantly higher intra-abdominal and lower subcutaneous adipose tissues compared to term-born infants at term-equivalent age, with illness severity as the principal predictor of intra-abdominal adiposity resulting in altered adipose tissue partitioning.^38^ Taken together, these findings highlight the importance of body composition in providing a more accurate assessment of growth status, especially in preterm infants. Although not exclusively in a preterm infant population, body composition curves illustrate that body composition is dynamic during infancy, which may not be reflected by traditional anthropometric growth measures.^23^

Strengths of our study include the use of a large longitudinal cohort of preterm infants born in a tertiary center. This comprehensive approach allowed us to investigate the association between HCA and multiple growth outcomes, including FM accretion. We also performed a mediation analysis to study the indirect effects of preterm birth on growth outcomes.

Our study also has some limitations. First, this was a retrospective cohort study, so we are unable to determine a causal relationship between HCA severity and FM accretion. Second, the etiology of HCA could not be determined. HCA and FIR can also result from sterile intraamniotic inflammation triggered by damage-associated molecular patterns related to tissue injury, hypoxemia, hemorrhage, and meconium, all of which might influence FM accretion and were not accounted for in this study. Third, information on infant NICU course, including antibiotic use, positive blood cultures, and nutritional intake, was limited. Fourth, our study only included preterm infants with body composition analysis performed using ADP, which measures total FM but does not provide information on fat distribution. Because HCA is uncommon in term infants and placental pathology is rarely performed for them, our findings are not generalizable to term infants. Furthermore, due to the impracticalities of placing infants into a separate air chamber,^39^ ADP is limited to clinically stable preterm infants. Thus, it is possible that our findings predicted an association that is weaker than the actual relationship, assuming that more infants exposed to HCA were unable to undergo ADP assessment. Another limitation is that mother-infant dyads were only recruited from a single referral academic hospital, which may limit the generalizability of our findings. Our mediation analysis indicated a direct association between HCA and an increase in FM *z* score at term-equivalent age, independent of gestational age. However, it is inconclusive whether HCA was directly associated with FM accretion in general, as non-significant direct effects were observed for %BF and %BF *z* score. Additional data is required to examine these questions as the current sample is likely underpowered. Future research should investigate the long-term effects of HCA, particularly FIR, on clinical endpoints such as brain development and biomarkers such as blood sugars, glucose to insulin ratio, insulin use, and cortisol levels.

## Conclusion

To the best of our knowledge, this is the first study reporting the direct dose-dependent effect of HCA severity on FM *z* score at term-equivalent age. Preterm birth accounted for only half of the FM *z* score and the remaining half was the direct adverse effect of HCA on FM *z* score. Our findings indicate that accurate assessment of body composition in preterm infants requires consideration of HCA as well, because HCA may partially explain rapid FM accretion as a potential adaptive mechanism in preterm infants. Future studies attempting to analyze causes of rapid FM accretion in preterm infants should include information on HCA either through placental pathology reports or postnatal biomarkers, including cytokine profiles. Additional research could further explore the associations between HCA and FM distributions as well as other body composition models. Further studies should also extend follow-up periods to older ages to provide comprehensive growth profiles and measure metabolic biomarkers.

## Supplementary information

Supplemental Materials

## Data Availability

The datasets generated for the current study are available from the corresponding author on reasonable request.
