# Supplementary material for: Histologic chorioamnionitis and fat mass accretion in infants born preterm
Source: Pediatr Res. 2025 Sep 19;99(4):1443–50. doi: 10.1038/s41390-025-04413-2 (PMC13102683; doi:10.1038/s41390-025-04413-2)
Supplement: Supplementary file 1 — Supplemental Materials [file 41390_2025_4413_MOESM1_ESM.pdf]

Supplement Table 1. Growth outcomes by maternal inflammatory response (MIR) excluding growth-restricted infants

	Total (n=341)	MIR None (n=238)	MIR Stage 1 (n=19)	MIR Stage 2 (n=57)	MIR Stage 3 (n=27)	*p*-value
**Body Composition at term-equivalent age***						
Fat Mass, kg	0.38 (0.25-0.46)‡	0.32 (0.24-0.43)‡	0.34 (0.23-0.46)‡	0.37 (0.30-0.46)‡	0.46 (0.35-0.66)‡	**<0.0001**
Body Fat Percentage, %	15.0 ± 4.9†	14.4 ± 4.9†	14.0 ± 5.5†	16.3 ± 4.1†	18.5 ± 4.4†	**<0.0001**
Fat Mass z score	0.87 ± 1.09†	0.72 ± 1.11†	0.84 ± 1.11†	1.23 ± 0.90†	1.43 ± 0.94†	**0.0004**
Body Fat Percentage z score	1.41 ± 1.18†	1.26 ± 1.19†	1.17 ± 1.27†	1.79 ± 1.01†	2.14 ± 0.97†	**0.0001**
Mid-Upper Arm Circumference, cm	8.8 (8.0-9.4)‡	8.7 (8.0-9.2)‡	8.8 (8.3-9.0)‡	8.8 (8.1-9.3)‡	10.0 (9.0-10.3)‡	0.0582
**Anthropometric Growth Rates****						
Change in Weight z score	-0.94 ± 0.59†	-0.89 ± 0.54†	-0.91 ± 0.59†	-1.01 ± 0.58†	-1.28 ± 0.87†	**0.0092**
Change in Length z score	-1.29 ± 0.94†	-1.21 ± 0.85†	-1.36 ± 1.10†	-1.44 ± 0.97†	-1.58 ± 1.41†	0.1355
Change in Head Circumference z score	-0.85 (-1.40 to -0.26)‡	-0.84 (-1.42 to -0.25)‡	-0.65 (-1.01 to 0.17)‡	-0.75 (-1.30 to -0.18)‡	-1.05 (-1.79 to -0.70)	0.0929
Weight gain, g/day	21 (17-24)‡	21 (17-24)‡	23 (14-26)‡	22 (17-25)‡	19 (15-23)‡	0.6289
Weight gain (2-point), g/kg/day	12 (10-14)‡	12 (10-14)‡	12 (10-14)‡	12 (11-14)‡	13 (12-14)‡	0.5378
Weight gain (Exponential), g/kg/day	13 (10-15)‡	13 (10-15)‡	12 (10-14)‡	13 (12-15)‡	14 (13-15)‡	0.1347
Length gain, cm/week	0.8 (0.6-1.1)‡	0.8 (0.6-1.0)‡	0.8 (0.3-1.0)‡	0.8 (0.6-1.1)‡	0.9 (0.6-1.1)‡	0.8480
Head Circumference gain, cm/week	0.7 (0.5-0.8)‡	0.7 (0.5-0.8)‡	0.6 (0.6-0.9)‡	0.7 (0.6-0.8)‡	0.7 (0.7-0.8)‡	0.0888

† Mean ± SD

‡ Median (IQR)

* Measured at 36 weeks post-menstrual age (PMA) or discharge

** Calculated between birth and 36 weeks PMA or discharge

Supplement Table 2. Growth outcomes by fetal inflammatory response (FIR) excluding growth-restricted infants

	Total (n=337)	FIR None (n=254)	FIR Stage 1 (n=29)	FIR Stage 2 (n=39)	FIR Stage 3 (n=15)	*p*-value
**Body Composition at term-equivalent age***						
Fat Mass, kg	0.33 (0.25-0.45)‡	0.33 (0.24-0.43)‡	0.39 (0.29-0.55)‡	0.40 (0.32-0.50)‡	0.33 (0.29-0.46)‡	**0.0112**
Body Fat Percentage, %	15.2 (12.3-17.5)‡	14.8 (11.9-17.2)‡	16.8 (13.4-19.3)‡	16.6 (14.3-19.7)‡	15.2 (12.7-18.3)‡	**0.0136**
Fat Mass z score	0.86 ± 1.10†	0.75 ± 1.10†	1.18 ± 1.16†	1.21 ± 0.96†	1.23 ± 0.80†	**0.0129**
Body Fat Percentage z score	1.40 ± 1.18†	1.29 ± 1.18†	1.77 ± 1.34†	1.76 ± 1.02†	1.76 ± 0.88†	**0.0156**
Mid-Upper Arm Circumference, cm	8.9 ± 1.3†	8.8 ± 1.4†	9.3 ± 1.3†	8.9 ± 1.1†	8.8 ± 0.9†	0.3193
**Anthropometric Growth Rates****						
Change in Weight z score	-0.95 ± 0.59†	-0.91 ± 0.56†	-1.10 ± 0.60†	-0.96 ± 0.72†	-1.19 ± 0.64†	0.1320
Change in Length z score	-1.28 ± 0.93†	-1.24 ± 0.89†	-1.75 ± 0.84†	-1.21 ± 1.12†	-1.26 ± 1.04†	**0.0394**
Change in Head Circumference z score	-0.82 ± 1.04†	-0.79 ± 1.05†	-0.90 ± 0.79†	-0.99 ± 1.21†	-0.87 ± 0.94†	0.6874
Weight gain, g/day	20 ± 7†	20 ± 7†	20 ± 7†	21 ± 7†	20 ± 5†	0.9207
Weight gain (2-point), g/kg/day	12 (10-14)‡	12 (10-14)‡	12 (11-13)‡	12 (12-15)‡	12 (10-13)‡	0.4390
Weight gain (Exponential), g/kg/day	13 (10-15)‡	13 (10-15)‡	13 (12-14)‡	14 (12-15)‡	13 (10-14)‡	0.2157
Length gain, cm/week	0.8 ± 0.4†	0.8 ± 0.4†	0.8 ± 0.3†	0.8 ± 0.7†	0.8 ± 0.4†	0.9237
Head Circumference gain, cm/week	0.7 (0.5-0.8)‡	0.7 (0.5-0.8)‡	0.7 (0.6-0.8)‡	0.7 (0.6-0.8)‡	0.7 (0.6-0.9)‡	0.2794

† Mean ± SD

‡ Median (IQR)

* Measured at 36 weeks post-menstrual age (PMA) or discharge

** Calculated between birth and 36 weeks PMA or discharge

Supplement Table 3. Mediation analysis measuring the direct effect of maternal inflammatory response (MIR) and indirect effect of gestational age on growth outcomes

	**Total effect**[†]				**Direct effect**[†]				**Indirect effect**[†]				**% Mediated**[‡]
	**β**	**95% CI**	**Bootstrap 95% CI**	***p*-value**	**β**	**95% CI**	**Bootstrap 95% CI**	***p*-value**	**β**	**95% CI**	**Bootstrap 95% CI**	***p*-value**	
**Body Composition at term-equivalent age***													
Body Fat Percentage, %	1.15	0.62-1.68	0.64-1.66	**<0.0001**	0.14	-0.33 to 0.62	-0.35 to 0.64	0.5576	1.01	0.68-1.33	0.66-1.36	**<0.0001**	87.6
Fat Mass z score	0.24	0.12-0.36	0.12-0.37	**<0.0001**	0.12	0.01-0.25	-0.03 to 0.28	**0.0463**	0.12	0.07-0.17	0.05-0.19	**<0.0001**	49.1
Body Fat Percentage z score	0.29	0.16-0.42	0.16-0.42	**<0.0001**	0.12	-0.01 to 0.25	-0.02 to 0.27	0.0602	0.17	0.11-0.24	0.09-0.25	**<0.0001**	58.4
**Anthropometric Growth Rate***													
Change in Weight z score	-0.09	-0.15 to -0.02	-0.17 to -0.01	**0.0084**	-0.04	-0.10 to 0.03	-0.12 to 0.05	0.3000	-0.05	-0.08 to -0.02	-0.08 to -0.02	**0.0002**	59.2

[†] Adjusted for delivery mode, maternal BMI at hospital admission ≥ 30.0 kg/m^2, and hypertension
[‡] Calculated by dividing the indirect effect by total effect
* Measured at 36 weeks post-menstrual age (PMA) or discharge
** Calculated between birth and 36 weeks PMA or discharge

Supplement Table 4. Mediation analysis measuring the direct effect of fetal inflammatory response (FIR) and indirect effect of gestational age on growth outcomes

	Total effect†				Direct effect†				Indirect effect†				% Mediated‡
	**β**	**95% CI**	**Bootstrap 95% CI**	***p*-value**	**β**	**95% CI**	**Bootstrap 95% CI**	***p*-value**	**β**	**95% CI**	**Bootstrap 95% CI**	***p*-value**	
**Body Composition at term-equivalent age***													
Body Fat Percentage, %	0.83	0.22-1.47	0.22-1.46	**0.0077**	0.19	-0.33 to 0.72	-0.34 to 0.72	0.4712	0.65	0.29-1.01	0.26-1.04	**0.0004**	77.0
Fat Mass z score	0.20	0.06-0.34	0.07-0.34	**0.0047**	0.12	-0.02 to 0.26	-0.02 to 0.26	0.0827	0.08	0.03-0.13	0.02-0.14	**0.0015**	40.6
Body Fat Percentage z score	0.23	0.08-0.38	0.09-0.37	**0.0029**	0.12	-0.03 to 0.26	-0.03 to 0.26	0.1079	0.11	0.05-0.18	0.03-0.19	**0.0007**	49.6

† Adjusted for delivery mode, maternal BMI at hospital admission ≥ 30.0 kg/m^2, and hypertension

‡ Calculated by dividing the indirect effect by total effect

* Measured at 36 weeks post-menstrual age (PMA) or discharge

** Calculated between birth and 36 weeks PMA or discharge
